# Patient Demographics in Acute Care Surgery at the Ruijin Hospital in Shanghai

**DOI:** 10.5402/2011/801404

**Published:** 2011-07-07

**Authors:** Willem Folmer, Wim Lammers, Terry Mulligan, Esther M. M. Van Lieshout, Peter Patka, Zhenye Xu, Yiming Lu, Dennis Den Hartog

**Affiliations:** ^1^Department of Surgery-Traumatology, Erasmus MC, University Medical Center Rotterdam, P.O. Box 2040, 3000 CA Rotterdam, The Netherlands; ^2^Department of Emergency Medicine, Erasmus MC, University Medical Center Rotterdam, P.O. Box 2040, 3000 CA Rotterdam, The Netherlands; ^3^Department of Emergency Medicine and Acute Surgery-Traumatology, Ruijin Hospital, Shanghai Jiao Tong University School of Medicine, No. 197 Rui Jin Er Road, Shanghai 200025, China

## Abstract

Acute Care Surgery is a discipline that includes trauma care, surgical critical care, and emergency surgery. It is organized in different models and provides mainly operative and nonoperative care. The aim of this study was to provide a demographic analysis of the care of surgical patients at the Emergency Department (ED) in a large teaching hospital in Shanghai, where general surgeons and orthopedic surgeons take care for most of all acute surgery. A bilingual questionnaire was developed to collect data for patients referred to the general or orthopedic surgeon in the ED (June–September 2008). Data about the gender, age, diagnosis, diagnostic tools, treatments, and outcomes were collected. A total of 255 questionnaires were collected; the most common diagnoses of patients were infections of abdominal organs and fractures. Complementary diagnostics like X-ray (59%), blood tests (36%), and ultrasound (17%) were frequently used. More than half of the patients were discharged afterwards most of them with followup. This study gives a first overview of acute care surgery of the emergency patients of the ED in a large Chinese metropolitan hospital.

## 1. Introduction

The first civilian trauma centres were built in the United States in the late 1960s. It was the revival of a surgical discipline that cares for the injured patients [1, 2]. In the 1980s trauma surgery became a specialty in the USA and in many places in Europe [3]. In this first period this specialty mainly supplied operative care, but nowadays the elective procedures are distinguished from trauma surgery and so the acute care surgery mainly provides nonoperative care [1]. Moreover, acute care surgery has become divided into many different specialties. This obscurity about the focus of acute care surgery has lead to an identity crisis of the discipline of trauma surgery [2, 3]. The acute care surgery evolved into trauma care, surgical critical care, and emergency surgery [4, 5], as described by the American Association for the Surgery of Trauma [6, 7]. Basically, all over the world there are two different models in which the acute care surgery is organised [8]. In one model emergency surgery is provided by an acute care surgery specialists including trauma surgeons and in another emergency surgery is subdivided among the different surgical specialties. The first model is mainly common in the United States; the second model predominates in Europe [9].

Little is known about this new specialty of acute care surgery outside Europe and the US. In China, where Emergency Medicine is a young discipline [10] and where also acute care surgery is still developing, the different Emergency Departments (EDs) vary much in the way the Emergency Medicine is organized. There is little numerical information concerning the patient demographics or the staffing patterns in the EDs [11]. At the Ruijin Hospital in Shanghai, acute care surgery is in many ways comparable to the European model. Although the European model is not a uniform system [9], physicians are committed to take calls on the basis of a duty system in many European countries. The same applies to the Ruijin Hospital. In the Ruijin Hospital, acute care surgery is not yet a full specialty. Except for thoracic and neurosurgery emergencies, the orthopedic and general surgeons provide mostly acute care surgery: the orthopedic surgeons treat the skeletal trauma, and the general surgeons treat the abdominal emergencies as well as visceral and skin injuries. 

In Shanghai, the ambulances are staffed with prehospital care providers, mainly doctors with varying degrees and levels of training. After arrival at the ED, surgical patients are admitted to the rooms of the general and orthopedic surgeon. Their close proximity allows for a quick and easy referral between general and orthopedic surgeons. An operating theatre is available for the minor surgery in the ED. 

The aim of this prospective study was to make a demographic analysis of the patients seen by general and orthopedic surgeons at the ED of Ruijin Hospital, in order to gain better insight into their acute surgery care organization. Data like gender, age, diagnosis, diagnostic tools, treatments, and outcomes of the patients were collected.

## 2. Materials and Methods

The current study was performed from June 5 to June 27 and from August 7 to September 11, 2008, at the ED of the Ruijin Hospital. The Ruijin Hospital is the largest teaching hospital in Shanghai, China. It has 1800 beds and is affiliated to Shanghai Jiao Tong University School of Medicine.

A questionnaire was developed for collecting data of the emergency patients who visited an orthopedic or a general surgeon at the ED. Questions for these patients included gender, date of birth, race, date of visit, time of visit, specialist (orthopedic surgeon or general surgeon), diagnosis (31 items and a free text field for diagnoses not listed), diagnostic tests performed (eight items), procedures performed (13 items), medication prescribed (nine items, including Traditional Chinese Medication), vital signs (heart rate, temperature, blood pressure, respiratory rate, and PaO_2_), and outcome (seven items) (see Table 1(a) and 1(b)). In the case of trauma patients, the type of trauma (blunt, penetrating, or burn) and the anatomic location (head, chest, abdomen, extremities, spine, or other) were also recorded. The questionnaires were completed by the orthopedic or general surgeons as well as by medical students who did their internships at this department. 

**Figure 1:**
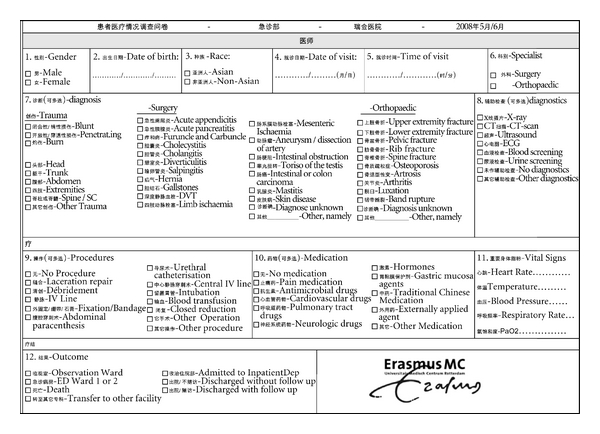
Study questionnaire.

The questionnaire was developed with the help of local staff members of the ED. Chinese medical specialists and medical students also did the translation into Chinese. Initial translations were verified by another group of the medical staff and adjusted where needed until all involved considered it perfect. The final questionnaire was bilingual, combining both the English and Chinese text (Figure 1).

Data were entered into a database and analyzed using the Statistical Package for the Social Sciences version 12.0 (SPSS, Chicago, Ill, USA). Frequencies were calculated for all items of the questionnaire. Age was presented as median with 1st and 3rd quartile. 

## 3. Results

During the study period 255 questionnaires were completed at the surgery rooms of the ED, 152 by general surgeons and 102 by orthopedic surgeons. For one patient it was unknown which specialist was consulted. The patients had a median age of 50 years (P_25_–P_75_ 34–66 years). Patients were almost exclusively Asian, and a slight majority was male. A demographic description of this study population is given in Table 1. Percentages are given excluding the unknowns.

### 3.1. Diagnosis

At the general surgery room, trauma was the most commonly registered diagnosis (20.3%), followed by cholecystitis (18.2%), pancreatitis (17.6%), and appendicitis (11.5%). At the orthopedic surgery room, trauma accounted for 77.5% of all diagnoses, followed by upper extremity fractures (21.4%). 

Of all fractures 47% were located in the upper extremities and 37% were located in the lower extremities. The remaining fractures were located in the pelvis, rib, or spine. Other than fractures, soft-tissue injuries (19.4%) and tendon/ligament ruptures (11.2%) were the most commonly registered diagnoses of the orthopedic patients. 

### 3.2. Trauma Type and Location

As mentioned above, 109 patients presented to the surgery room with traumatic injuries. The general surgeon treated most of the trauma patients with penetrating trauma (76%), while almost all the blunt-trauma patients (92%) were referred to the orthopedic surgeon. Two patients seen by the general surgeon had both blunt and penetrating injuries. No patients with burn wounds were treated at the ED during the study period, because these patients were generally admitted to emergency room of the Burn Center of the Ruijin Hospital. Most of the trauma patients had injuries in the extremities (68.1%); the orthopedic surgeon diagnosed 70% of them. 

### 3.3. Diagnostics

A diagnostic test was performed in 129 out of 152 patients. The general surgeons used mainly blood test (62%), X-ray (31%), or ultrasound (29%). Orthopedic surgeons requested X-rays for almost every patient (97%). They rarely ordered any other type of diagnostic test. 

### 3.4. Procedures

In 57% of cases, no intervention was required. In case an intervention was needed, general surgeons mainly performed debridement or laceration repair (22 out of 39 patients, excluding unknown). Orthopedic surgeons mainly applied fixation bandage (plaster or compression bandage; 28 out of 42 patients.

### 3.5. Medication

Thirty-two out of 152 patients seen by the general surgeon did not require any drugs; for another 15 patients data are lacking. Of the 105 patients that required drugs, 82 received antimicrobial drugs (78.1%), 24 received painkillers (22.8%), and 19 received gastric medication (18.1%). Orthopedic surgeons administered drugs to 79 patients (93.0%), of which 55 patients received pain medication (70.0%). Antimicrobial drugs and Traditional Chinese Medication were administered to nine and eight patients, respectively.

### 3.6. Outcome

Almost half of the patients referred to a general surgeon were discharged (84.8% with followup and 15.2% without). A substantial number of patients were sent to the observation unit of ED (*N* = 42). The other patients were transferred to another doctor or facility (*N* = 14), to the inpatient department for further treatment (*N* = 14), or to the acute surgery and trauma ward of the ED (*N* = 4).

The orthopedic surgeon discharged 65 of his patients (79.3%); 55 patients with followup and 10 without. Fourteen patients were admitted to the inpatient department, two patients were sent to another doctor or another facility, and one patient was sent to the observation unit. 

### 3.7. Most Common Diagnosis

A subanalysis of the most common diagnoses (trauma, cholecystitis, pancreatitis, appendicitis, and extremities fractures) made by the general and orthopedic surgeon is shown in Table 2. Of all the patients, 93% suffered from any of these injuries or conditions. An X-ray was performed in 77% of trauma patients. Almost 40% of these patients did not receive any intervention; however, 70 out of 109 patients received pain killers or antimicrobial medication. Most patients were discharged, while 14 patients were sent to the inpatient relative department. The most commonly requested diagnostic tool used for patients with cholecystitis, pancreatitis, or appendicitis was a blood test. Of these patients, 81%, 74%, and 65% were treated with antimicrobial drugs, respectively. Although most patients with cholecystitis were sent home, most patients with pancreatitis were admitted for observation for a longer time in the ED. Most patients with suspected appendicitis were either discharged or sent to the observation unit of ED. Soft-tissue injury represented 19% of the diagnoses of the orthopedic surgeon. In most cases this diagnosis was made after reading the X-ray. Of the patients with soft-tissue injury, 88% did not receive any intervention. Almost half of the patients received pain medication. All patients with a soft-tissue injury were discharged, most of them with a follow-up control. 

Almost all fractures were confirmed on X-ray; CT scan was made only once. Fifty percent of the patients with lower-extremity fractures and 65% of the patients with upper-extremity fractures were treated with a fixation bandage. Most often they received painkillers, six of them received traditional Chinese medication. Approximately 25% of these patients did not receive any medication at all. Fourteen patients (39%) were admitted after treatment; the others were generally sent home with a request for followup. 

## 4. Discussion

This study provides a demographic analysis of the acute care surgery of Ruijin Hospital, which is organised according to the European multidisciplinary model. Orthopedic and general surgeons provide most of the emergency surgery care. During the study period, patients were almost exclusively diagnosed with trauma, cholecystitis, pancreatitis, appendicitis, or soft-tissue injury.

A relatively high number of patients did not require interventions and were sent home without further followup. This implies that patients referred to a general surgeon or orthopedic surgeon were only mildly injured, if injured at all. Some patients received pain medication. This is due to the fact that, unlike many western countries, China does not have a general practitioner system. This was also concluded from a parallel study performed at the Ruijin Hospital during the same study period, revealing that only 4.5% of the patients were transferred to the hospital by ambulance [12]. Procedures performed were mostly restricted to soft tissue repair, debridement, or a fixation bandage. Overall, most of the emergency surgery care consists of nonoperative care. 

Examination and treatment of trauma patients covers 43% of the work done by general and orthopedic surgeons. Of the trauma patients, those with penetrating trauma were referred to the general surgeon, and the orthopedic surgeon mostly treated those with blunt force trauma. No burn injuries were reported during the study period at the ED, since the Ruijin Hospital has a large Burn Center with a separate emergency room. Many trauma patients were not treated extensively, implying that injury severity was limited. One reason for that is probably that the Ruijin Hospital is situated in the centre of Shanghai, where traffic speeds tend to be limited.

A surprising observation is the high prescription rate of antimicrobial drugs. The general surgeons administered this type of drugs in 54% of cases. It is unknown whether or not this practice results in high rates of resistance against antimicrobial agents; it would be interesting and useful to do more research on this subject.

## 5. Conclusion

There are only few published studies about the acute care surgery in China. This study gives a broad general overview of the care of the surgical emergency patients of the Emergency Department and so better understandings of the working area of the acute care surgery at the Ruijin Hospital, a teaching hospital in the center of Shanghai.

## Figures and Tables

**Table:** (a)

		Total		General surgery		Orthopedic surgery	
		*N = *255	*%*	*N = *152	*%*	*N = *102	*%*
Gender	Male	125	49.0	75	49.3	50	49.0
	Female	117	45.9	66	43.4	50	49.0
	Unknown	13	5.1	11	7.2	2	2.0

Race	Asian	250	98.0	150	98.7	99	97.1
	Non-Asian	3	1.2	1	0.7	2	2.0
	Unknown	2	0.8	1	0.7	1	1.0

Diagnosis^1^	Trauma	109	42.7	30	19.7	79	77.5
	Appendicitis	17	6.7	17	11.2	0	0.0
	Pancreatitis	27	10.6	26	17.1	0	0.0
	Furuncle/carbuncle	1	0.4	1	0.7	0	0.0
	Cholecystitis	27	10.6	27	17.8	0	0.0
	Cholangitis	3	1.2	3	2.0	0	0.0
	Hernia	3	1.2	2	1.3	1	1.0
	Gallstones	10	3.9	10	6.6	0	0.0
	Limb ischemia	1	0.4	1	0.7	0	0.0
	Mesenteric ischemia	1	0.4	1	0.7	0	0.0
	Intestinal carcinoma	1	0.4	1	0.7	0	0.0
	Intestinal obstruction	8	3.1	8	5.3	0	0.0
	Skin disease	3	1.2	3	2.0	0	0.0
	General surgical disease, diagnosis unkown^2^	8	3.1	8	5.3	0	0.0
	Other surgery	25	9.8	25	16.4	0	0.0
	Upper extremity fracture	23	9.0	2	1.3	21	20.6
	Lower extremity fracture	18	7.1	1	0.7	17	16.7
	Pelvic eracture	1	0.4	0	0.0	1	1.0
	Rib fracture	3	1.2	1	0.7	2	2.0
	Spine fracture	4	1.6	0	0.0	4	3.9
	Osteoporosis	2	0.8	0	0.0	2	2.0
	Arthrosis	1	0.4	0	0.0	1	1.0
	Arthritis	1	0.4	0	0.0	1	1.0
	Luxation	1	0.4	0	0.0	1	1.0
	Band rupture	11	4.3	0	0.0	11	10.8
	Other orthopedics	35	13.7	0	0.0	35	34.3
	Unknown^3^	8	3.1	4	2.6	4	3.9

Other Diagnoses	Abscess	2	0.8	2	1.3	0	0.0
	Fecal obstruction	1	0.4	1	0.7	0	0.0
	Fever after cholecystectomy	1	0.4	1	0.7	0	0.0
	Liver mass	1	0.4	1	0.7	0	0.0
	Liver sclerosis	1	0.4	1	0.7	0	0.0
	Pain after appendectomy	1	0.4	1	0.7	0	0.0
	Spleen rupture	1	0.4	1	0.7	0	0.0
	Pyelonephritis	1	0.4	1	0.7	0	0.0
	Urinary stone	2	0.8	2	1.3	0	0.0
	Cancer	6	2.4	7	3.9	0	0.0
	Other general surgery	6	2.4	6	3.9	0	0.0
	Skin infection	3	1.2	2	1.3	1	1.0
	Muscle pain	6	2.4	0	0.0	6	5.9
	Shoulder dislocation	1	0.4	0	0.0	1	1.0
	Soft-tissue injury	19	7.5	0	0.0	19	18.6
	Other orthopedic surgery	4	3.1	0	0.0	8	7.8

Trauma type	Blunt	48	44.0	4	13.3	44	55.7
	Penetrating	25	22.9	19	63.3	6	7.6
	Burn	0	0.0	0	0.0	0	0.0
	Unknown	38	34.9	9	30.0	29	36.7

Anatomic location	Head	12	11.0	9	30.0	3	3.8
	Trunk	14	12.8	1	3.3	13	16.5
	Abdomen	4	3.7	2	6.7	2	2.5
	Extremities	62	56.9	18	60.0	44	55.7
	Spine and spinal cord	2	1.8	0	0.0	2	2.5
	Other trauma	5	4.6	3	10.0	2	2.5
	Unknown	18	16.5	1	3.3	16	20.3

^1^Deep vein thrombosis, aneurysm/dissection of artery, torsio testis, and mastitis were also listed but not diagnosed in any of the patients.

^2^Diagnosis unknown for specialist.

^3^Diagnosis not completed for these patients.

**Table:** (b)

		Total		General surgery		Orthopedic surgery	
		*N = *255	%	*N = *152	%	*N = *102	%

Diagnostics	X-ray	129	50.6	38	25.0	91	89.2
	CT	33	12.9	29	19.1	4	3.9
	Ultrasound	37	14.5	36	23.7	0	0.0
	ECG	13	5.1	11	7.2	2	2.0
	Blood	78	30.6	76	50	1	1.0
	Urine	25	9.8	25	16.4	0	0.0
	Other diagnostics	0	0.0	0	0.0	0	0.0
	None	24	9.4	23	15.1	1	1.0
	Unknown	37	14.5	29	19.1	8	7.8

Procedure	No procedure	105	41.2	57	37.5	48	47.1
	Laceration repair	21	8.2	15	9.9	6	5.9
	Debridement	27	10.6	17	11.2	10	9.8
	IV line	9	3.5	8	5.3	1	1.0
	Fixation bandage	32	12.5	4	2.6	28	27.5
	Catheterisation	1	0.4	0	0.0	1	1.0
	Central IV line	2	0.8	2	1.3	0	0.0
	Intubation	2	0.8	2	1.3	0	0.0
	Abdominal Paracentesis	0	0.0	0	0.0	0	0.0
	Blood transfusion	1	0.4	0	0.0	1	1.0
	Close reduction	5	2.0	0	0.0	5	4.9
	Other operation	11	4.3	6	3.9	5	4.9
	Other procedure	0	0.0	0	0.0	0	0.0
	Procedure unknown	69	27.1	56	36.8	12	11.8

Medication	No medication	55	21.6	32	21.1	23	22.5
	Pain medication	80	31.4	24	15.8	55	53.9
	Antimicrobial drugs	92	36.1	82	53.9	9	8.8
	Cardiovascular drugs	1	0.4	1	0.7	0	0.0
	Pulmonary drugs	0	0.0	0	0.0	0	0.0
	Neurological drugs	0	0.0	0	0.0	0	0.0
	Hormones	1	0.4	1	0.7	0	0.0
	Gastric mucose agents	20	7.8	19	12.5	0	0.0
	Traditional Chinese Medicine	11	4.3	3	2.0	8	7.8
	Externally applied agent	8	3.1	5	3.3	3	2.9
	Other medication	44	17.3	41	27	3	2.9
	Medication unknown	32	12.5	15	9.9	17	16.7

Outcome	Observation ward	44	17.3	42	27.6	1	1.0
	ED ward 1 or 2	4	1.6	4	2.6	0	0.0
	Transfer other doctor/facility	16	6.3	14	9.2	2	2.0
	Admitted to Inpatient Dep.	28	11	14	9.2	14	13.7
	Discharged without folloup	20	7.8	10	6.6	10	9.8
	Discharged with follow up	111	43.5	56	36.8	55	53.9
	Outcome Unknown	32	12.5	12	7.9	20	19.6

**Table 2:** Diagnostics, procedure, medication, and outcome of the most common diagnoses.

		Trauma	Chol*	Panc*	App*	STI*	UEF*	LEF*
		*N = *109	*N = *27	*N = *27	*N = *17	*N = *19	*N = *23	*N = *18

Diagnostics	X-ray	84	3	6	5	17	23	16
	CT	9	2	10	4	0	0	1
	Ultrasound	2	9	9	4	0	0	0
	ECG	4	3	2	1	0	0	3
	Blood	2	17	22	13	0	0	1
	Urine	0	7	9	3	0	0	0
	None	9	2	3	1	0	0	0
	Other	0	0	0	0	0	0	0
	Unknown	11	7	2	3	2	0	1

Procedure	No procedure	43	7	9	8	14	4	5
	Laceration repair	21	0	0	0	0	2	2
	Debridement	26	0	0	0	1	2	5
	IV line	1	2	5	2	0	0	0
	Fixation bandage	23	0	0	0	2	15	9
	Abdom. paracenthesis	0	0	0	0	0	0	0
	Catheterisation	1	0	0	0	0	0	1
	Central IV line	0	0	1	0	0	0	0
	Intubation	0	0	2	0	0	0	0
	Blood transfusion	1	0	0	0	0	0	1
	Close reduction	4	0	0	0	0	3	0
	Other operation	3	1	1	1	0	4	1
	Other procedure	0	0	0	0	0	0	0
	Procedure unknown	10	17	11	6	3	0	0

Medication	No medication	29	1	2	2	9	6	2
	Pain medication	50	6	9	1	8	12	11
	Antimicrobial drugs	20	22	20	11	2	1	3
	Cardiovascular drugs	0	0	1	0	0	0	0
	Pulmonary drugs	0	0	0	0	0	0	0
	Neurological drugs	0	0	0	0	0	0	0
	Hormones	0	0	0	0	0	0	0
	Gastric mucose agents	0	2	14	1	0	0	0
	TCM	4	1	2	0	0	4	2
	Ext. applied agent	4	0	2	0	1	0	0
	Other medication	9	12	6	5	0	1	0
	Medication unknown	10	2	1	4	0	4	4

Outcome	Observation ward	3	7	16	6	0	0	1
	ED ward 1 or 2	0	1	0	0	0	0	0
	Transfer other facility	6	0	0	0	0	1	0
	Admitted to Inp. Dep.	14	1	3	1	0	8	6
	Discharged without followup	11	1	6	1	2	1	0
	Discharged with followup	57	14	0	7	12	11	8
	Death	0	0	0	0	0	0	0
	Outcome unknown	18	3	2	2	5	2	3

*Chol: cholecystitis Panc: pancreatitis; App: appendicitis; STI: soft-tissue injury; LEF: lower extremity fracture; UEF: upper extremity fracture.
